# IL-6 helps weave the inflammatory web during acute coronary syndromes

**DOI:** 10.1172/JCI167670

**Published:** 2023-06-01

**Authors:** Tetsushi Nakao, Peter Libby

**Affiliations:** 1 Division of Cardiovascular Medicine, Department of Medicine, Brigham and Women’s Hospital, Harvard Medical School Boston, Massachusetts, USA.; 2 Broad Institute, Cambridge, Cardiovascular Research Center, Massachusetts General Hospital, Boston, Massachusetts, USA.; 3 Department of Medical Oncology, Dana-Farber Cancer Institute, Boston, Massachusetts, USA.; 4 Medical Population and Genetics Program, Broad Institute, Cambridge, Massachusetts, USA.

## Abstract

The cytokine IL-6 has well-known proinflammatory roles in aging and ischemic heart disease. In this issue of the *JCI*, Alter and colleagues used mouse experiments and human tissue to investigate the source of IL-6 following myocardial infarction. The authors showed that cardiac fibroblasts produced IL-6 after coronary ligation in mice and proposed the existence of a pathway involving adenosine signaling via the adenosine A2b receptor. The findings underscore the complexity of IL-6 biology in ischemic heart disease and identify an adenosine/IL-6 pathway that warrants consideration for targeting as a modulator of cardiovascular risk.

## IL-6 signaling

**Figure 1:**
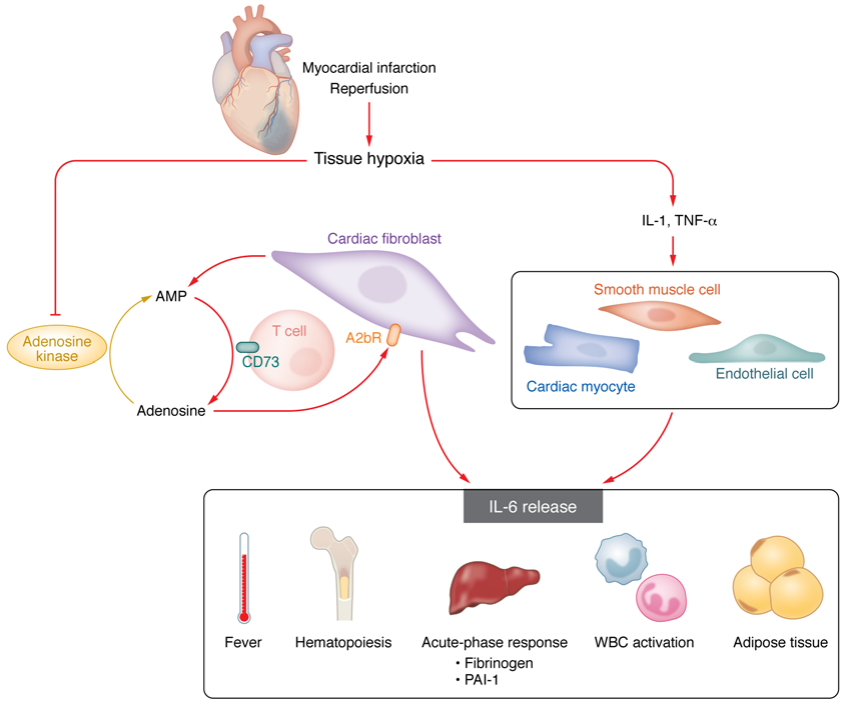
IL-6 is a multifaceted mediator of cardiovascular disease. Hypoxia in the ischemic region during an acute coronary syndrome leads to release of the nucleotide AMP. T cell–produced CD73 hydrolyzes this nucleotide to adenosine, which in turn engages the A2bR, which is predominantly expressed in the ischemic myocardium by cardiac fibroblasts. The cardiac fibroblasts respond by releasing IL-6. IL-1, also activated within the ischemic region, can impinge on cardiomyocytes, endothelial cells, and smooth muscle cells, as well as on resident macrophages, to augment local IL-6 production. IL-6 and IL-1 can mediate fever, a common concomitant of acute coronary syndromes. IL-6 can also stimulate hematopoiesis, contributing to the leukocytosis that can accompany acute coronary syndromes. Additionally, IL-6 can activate leukocytes and adipose tissue to augment local and systemic inflammation. In hepatocytes, IL-6 unleashes the acute-phase response, heightening the production of fibrinogen, the precursor of thrombi, and of plasminogen activator 1 (PAI-1), which inhibits endogenous fibrinolysis. A role for cardiac fibroblasts in producing IL-6 and orchestrating an inflammatory response during acute coronary syndrome extends our understanding of the complex circuits of inflammatory signaling following myocardial ischemic injury.

Many pathways converged with the identification of a cytokine now named IL-6. The activities now ascribed to IL-6 include B cell–stimulatory factor 2, hepatocyte-stimulating factor, 26 kDa protein, hybridoma-plasmacytoma growth factor, and IFN-β2 (1). This cacophony of names already indicates the myriad actions of this cytokine. Many cells can produce IL-6, including mesenchymal cells, vascular endothelial cells, smooth muscle cells, and a variety of leukocytes, adipose tissue, and muscle (Figure 1). As a major mediator of the acute-phase response in hepatocytes and downstream of IL-1 and TNF, IL-6 has pleiotropic effects on tissues including bone marrow, muscle, adipose, and the heart (Figure 1). Moreover, IL-6 is the emblematic cytokine of the senescence-associated secretory phenotype that is strongly implicated in aging.

IL-6 signaling is complex. The classic pathway uses the transmembrane IL-6 receptor (CD126), which, together with gp130, transduces IL-6 signals in hepatocytes and leukocytes. This classical pathway may mediate the antiinflammatory effects of IL-6. Alternatively, IL-6 signals through the *trans* pathway, in which CD126 shed from cell surfaces can join with IL-6 in the fluid phase of blood and signal through gp130 expressed on the plasma membranes of many cells. The *trans* pathway purportedly mediates many of the proinflammatory actions of IL-6. Whether downstream of the classical or *trans*-signaling pathways, intracellular signaling by IL-6 involves phosphorylation of STAT3, which modulates transcriptional control of a variety of targets.

In the cardiovascular system, the acute-phase response elicited by IL-6 promotes thrombus formation and stability (2). IL-6 can induce angiotensin II, which can stimulate hypertrophy of cardiac myocytes. IL-6 signaling may promote experimental atherosclerosis and modulate healing of the infarcted myocardium, but consensus regarding these effects is lacking. Overall, in humans, the net actions of IL-6 promote ischemic heart disease and its complications. IL-6 in plasma predicts first-ever, as well as recurrent, cardiovascular events and chronic coronary artery disease. The degree of elevation of IL-6 portends a poorer prognosis after acute coronary syndromes. CANTOS (Canakinumab Anti-inflammatory Thrombosis Outcomes Study) involved the use of a monoclonal antibody to neutralize IL-1β, a strong inducer of IL-6, and showed a reduction in recurrent cardiovascular events (3). Indeed, the reduction in IL-6 produced by canakinumab treatment in this study correlated well with a reduction in clinical events (3). Several smaller studies have targeted the IL-6 receptor and shown favorable effects on biomarkers and some indices of myocardial salvage, notably in the ASSAIL-MI (ASSessing the Effect of Anti-IL-6 Treatment in Myocardial Infarction) study (4, 5). These various findings have heightened interest in the local production of IL-6 in the ischemic myocardium.

## Cardiac fibroblasts produce IL-6

In this issue of the *JCI*, Alter and colleagues elegantly implicated cardiac fibroblasts as a major source of IL-6 following coronary ligation in mice. (6) The study authors proposed a pathway that involved adenosine signaling to cardiac fibroblasts via the adenosine A2b receptor (A2bR) and provide supportive evidence from observations in human tissue.

The infarcting myocardium presents a perfect storm for the operation of this adenosine/IL-6 pathway. Hypoxia can increase the adenosine receptor A2bR and provoke AMP release from injured cells. Adenosine arises from hydrolysis of AMP predominantly by CD73 on T lymphocytes. Alter and authors acknowledge that IL-1β and TNF may also stimulate IL-6 production locally in the myocardium. Indeed, we showed that IL-1β blockade in experimental myocardial infarction in mice can limit the reduction of left ventricular function through a combination of direct effects on the myocardium and altered hematopoiesis boosted by the acute myocardial injury. (7) Moreover, following coronary artery ligation in mice, IL-6 produced by endothelial cells in the bone marrow hematopoietic niche can promote myelopoiesis that can drive atherosclerosis and potentiate myocardial injury. (8)

## Conclusions and future questions

Several questions remain for future research related to cardiac fibroblast–produced IL-6. How much of the influence of IL-6 on myocardial healing and the extracardiac milieu depends on classical versus *trans*-signaling? Alter and colleagues used *Acta2* as one marker to identify cardiac fibroblasts. As this actin isoform abounds in smooth muscle cells, and these cells can produce copious amounts of IL-6, could smooth muscle cells, in addition to cardiac fibroblasts, furnish IL-6 during myocardial infarction (9)?

The insights of Alter and colleagues underscore the complexity of IL-6 biology in ischemic heart disease. Given the conflicting experimental literature regarding atherosclerosis and effects on the myocardium ascribed to IL-6 and the complexity of IL-6 signaling, and in view of the varied actions of IL-6 based on the precise timing and location, large-scale clinical endpoint trials will be needed to determine the effects of IL-6 inhibition on cardiovascular events. Beyond ASSAIL-MI, the RESCUE study (NCT03926117; Trial to Evaluate Reduction in Inflammation in Patients with Advanced Chronic Renal Disease Utilizing Antibody-mediated IL-6 Inhibition) targeted IL-6 ligand in patients with chronic kidney disease at high risk for cardiovascular complications (10). This phase II study showed striking dose-dependent reductions in IL-6. The ZEUS study (NCT05021835; A Research Study to Look at How Ziltivekimab Works Compared to Placebo in People with Cardiovascular Disease, Chronic Kidney Disease and Inflammation), now underway, will determine the effects of IL-6 ligand inhibition on cardiovascular events in a similar population of individuals with chronic kidney disease (stage 3–5) and C-reactive protein (CRP) levels above the median, i.e., a high-sensitivity CRP (hsCRP) of greater than 2 mg/L, despite contemporary medical therapy. Ultimately, only large-scale clinical outcome trial results will help us sort out the net effects of interruption of IL-6 signaling in patients. IL-6 has emerged as an attractive therapeutic target for cardiovascular risk modulation, a hypothesis whose time for testing has come.

## Version 1 06/01/2023

Electronic publication
